# Editorial: Women in lipid and fatty acid research: 2022

**DOI:** 10.3389/fphys.2023.1233624

**Published:** 2023-09-27

**Authors:** Vincenza Cifarelli, Morgan Ross, Linda R. Peterson

**Affiliations:** ^1^ Department of Pharmacology and Physiology, Saint Louis University School of Medicine, St. Louis, MO, United States; ^2^ Division of Cardiology, Department of Medicine, Washington University School of Medicine, St. Louis, MO, United States

**Keywords:** lipid, cardiovascular, Framingham, dietary fat, liver

The Research Topic *“Women in Lipid and Fatty Acid Research: 2022”* highlights work led by women in the field of lipid physiology. Biases and gender stereotypes have historically excluded and/or discouraged women from STEM research, causing women in science to be underrepresented and/or underacknowledged in several STEM fields even today. In spite of this, women researchers continue to make crucial scientific advances. The Research Topic, comprised by a mini review, two original articles and a brief research report, covers some of the areas of research by women in lipid and fatty acid metabolism.

Lipids and their fatty acid components regulate cellular homeostasis via lipidation, a post-translation modification involving the attachment of a lipid group to target proteins. Lipidations, which include palmitoylation, myristoylation, and prenylation, regulate protein conformation, function, and stability; however, only palmitoylation, first reported in 1951 (Folch and Lees, 1951), is a reversible enzymatically regulated modification (Chen et al., 2018). The mini-review by Dennis and Heather, summarizes key concepts of the palmitoylation process including 1) membrane transport proteins and kinases involved in substrate (palmitate) uptake into cells; 2) the enzymes that regulate palmitoylation and depalmitoylation cycles; and the 3) recently discovered process of mitochondrial palmitoylation (Kathayat et al., 2018). The review also discusses accumulating evidence related to the role of palmitoylation in tissue metabolism.

The liver is a key regulator of tissue metabolism and fatty acid synthesis. Due to the increasing prevalence of chronic liver diseases, research is needed to understand how hepatic dysfunction may affect systemic levels of fatty acids, an important cellular source of energy, and key regulators of metabolism, inflammation, and signaling (Mika and Sledzinski, 2017). During an orthotopic liver transplantation, the treatment of choice for liver failure in patients with end-stage liver disease (O'Leary et al., 2008), patients remain temporarily without liver in their body (anhepatic phase). Hliwa et al. investigated serum lipid profile in eighteen end-stage liver disease patients before surgery and after the anhepatic phase. The authors reported that impaired liver function affects circulating fatty acid composition; levels of serum myristic and palmitic acids were significantly decreased while levels of very long-chain fatty acids were increased after the anhepatic phase. Further studies are needed to determine the kinetics of re-establishing physiological levels of circulating lipids following an orthotopic liver transplant and the impact of the abnormal lipid profile on metabolism, inflammation and signaling in key metabolic tissues.

In the second original article of this series, Yiannakou et al. examine whether restricting intake of total and saturated fat would prevent cardiovascular disease (CVD) due to the hypothesis that higher saturated fat intakes increase serum low-density lipoprotein cholesterol (LDL-C), thus increasing the risk of CVD (Forouhi et al., 2018). Yiannakou et al. evaluates sex-specific associations between dietary intakes of saturated *versus* unsaturated fats, and cardiometabolic risk factors in the Framingham Offspring Cohort who were the offspring of participants in the original Framingham Heart Study (About FHS | Framingham Heart Study). Perhaps counterintuitively, the investigators found that saturated and monosaturated fat intakes both tended to be favorably associated with triglycerides-(TG)-to-high-density lipoprotein (HDL) ratio in both women and men. Moreover, they found no evidence was found to support an adverse relationship between these dietary fats and several surrogate markers of cardiometabolic health.

In another investigation of plasma lipoproteins, Sarkar et al. investigate protein binding of low-density lipoprotein (LDL). Approximately 30%–40% of LDL particles are bound to plasma proprotein convertase subtilisin/kexin type-9 (PCSK9), a secreted protein that binds and mediates endo-lysosomal degradation of low-density lipoprotein receptor (LDLR), limiting plasma clearance of cholesterol-rich LDL particles in liver. Authors have previously shown (Sarkar et al., 2020) that a gain-of-function mutation (R496W) in the PCSK9 gene prevents LDL binding to LDLR *in vitro*, however, the molecular details of this interaction remain unclear. In this new original research article, Sarkar et al. report the mode of action of gain-of-function mutations previously identified in individuals with multiple familial hypercholesterolemia (FH) (Abifadel et al., 2003). The mutations are located in two distinct regions of PCSK9 localized either within the cysteine-histidine–rich domain module 1 (CM1) region or within the surface-exposed region in the PCSK9 prodomain. The study also identified the Ser-127 residue to serve as a regulatory component. The S127R mutation renders PCSK9 resistant to inhibition by LDL. These findings are clinically important not only to the individuals with FH, that is, mediated by these mutations, but they also improve our understanding of how LDL levels may/may not be modulated by new PCSK9 inhibitor medications.

In conclusion, current women-led investigations are contributing key findings to our understanding of lipid and fatty acid metabolism, which are important regulators of whole-body metabolism, inflammation, and signaling. However, more basic, translational, and clinical studies are needed to improve our understanding of their complex contribution to tissue homeostasis and cardiometabolic diseases.
